# Evaluation of Bronchiolitis Severity and Hospitalization Rates in Pediatric Patients in Developing Countries Using the Pediatric Respiratory Severity Score

**DOI:** 10.7759/cureus.81173

**Published:** 2025-03-25

**Authors:** Naila Kanwal, Muhammad Shahzad, Abdur Rehman, Atta U Haq, Rameen Khalid, Saqlain Ghazanfar, Umm E Aimen Minhas, Muhammad Ahmad Khalid, Abdulqadir J Nashwan

**Affiliations:** 1 Pediatrics, Fatima Memorial Hospital, Lahore, PAK; 2 Pediatrics, Government Mian Muhammad Nawaz Sharif Hospital, Lahore, PAK; 3 Otolaryngology - Head and Neck Surgery, Rawalpindi Medical University, Rawalpindi, PAK; 4 Pediatrics, Khawaja Muhammad Safdar Medical College, Sialkot, PAK; 5 Pediatrics, Rawalpindi Medical University, Rawalpindi, PAK; 6 Internal Medicine, Rawalpindi Medical University, Rawalpindi, PAK; 7 Pediatrics, Allama Iqbal Medical College, Lahore, PAK; 8 Nursing and Midwifery Research, Hamad Medical Corporation, Doha, QAT

**Keywords:** diagnosis & prognosis, hospital admission, pediatrics emergency, respiratory bronchiolitis-interstitial lung disease, viral bronchiolo-alveolitis

## Abstract

Background

Bronchiolitis is a prevalent respiratory illness in young children, often leading to hospitalization due to severe symptoms. The Pediatric Respiratory Severity Score (PRESS) is a tool developed to categorize bronchiolitis severity and assist in clinical decision-making. This study evaluates bronchiolitis severity and its correlation with hospitalization rates using the PRESS score.

Methods

This prospective descriptive study enrolled 82 children (ages, two months to two years) with diagnosed bronchiolitis. Severity was assessed using the PRESS score, categorized into mild (0-1), moderate (2-3), and severe (4-5) groups. Hospitalization was determined based on clinical presentation, six hours after admission. The association between PRESS scores and hospitalization outcomes across demographics was analyzed.

Results

The PRESS scores classified 33 (40.2%) cases as severe, 27 (32.9%) as moderate, and 22 (26.8%) as mild. Hospitalization rates were higher in children with severe PRESS scores (n = 31, or 63.3%) compared to moderate (n = 18, or 36.7%) and mild cases (n = 0, or 0%). Associations between PRESS scores and hospitalization across demographic categories support PRESS as a predictor of hospitalization.

Conclusion

In conclusion, our study suggests that PRESS is a useful tool for classifying the severity of bronchiolitis and identifying patients at increased risk of hospitalization. However, given the limitations of this study, the results should be interpreted with caution. Further research is needed to validate the PRESS score in diverse populations and across different healthcare settings, to establish its broader applicability.

## Introduction

Bronchiolitis is one of the most frequent severe lower respiratory tract infections in children, frequently presenting with tachypnea and wheezing, primarily due to viral infections [1]. Affecting 5.74 per 10,000 population, approximately 2%-3% of children require hospitalization within their first year of life [2]. The condition is especially prevalent during the colder winter season and is a leading cause of pediatric hospital admissions [3,4]. High-risk patient categories for severe bronchiolitis encompass infants younger than six weeks, premature infants, and, notably, patients who already have chronic lung disease, congenital heart defects, or immunodeficiencies [5].

Accurate bronchiolitis severity scoring aids clinical decision-making and supports standardization for research and trials [6-8]. Critical indicators of respiratory severity, such as respiratory rate, wheezing, and cyanosis, are critical in guiding treatment decisions to prevent respiratory failure. Despite numerous studies evaluating infection severity, few have proposed simple bedside assessment tools for early intervention, similar to the APGAR (Appearance, Pulse, Grimace response, Activity, Respiration) score used for neonates [9].

Research supports the utility of respiratory rate, wheezing, retractions, and SpO_2_ in assessing respiratory status in children, with feeding difficulties being a crucial indicator of disease severity. The Pediatric Respiratory Severity Score (PRESS), developed to assess respiratory infection severity in children, includes these components. The PRESS score is an internationally used, validated scale that categorizes severity into mild, moderate, and severe, aiding in clinical decision-making regarding the need for hospitalization. Recent studies demonstrate that patients with moderate to severe scores have significantly higher hospitalization rates, and severe cases require prolonged oxygen therapy [10]. PRESS also helps predict the clinical course and duration of in-hospital treatment, making it a valuable tool in pediatric respiratory care.

PRESS was selected for its simplicity and practicality in clinical settings. Unlike other validated scoring systems, such as the BROSJOD (Bronchiolitis Score of Sant Joan de Déu) or Respiratory Distress Assessment Instrument (RDAI), which may require specialized equipment or extensive assessment, PRESS offers a non-invasive, quick, and reliable method for stratifying bronchiolitis severity. This makes it highly applicable across various settings, including resource-limited environments, such as emergency wards, general wards, and ICUs [8,9].

Furthermore, PRESS incorporates critical indicators such as respiratory rate, wheezing, retractions, oxygen saturation, and feeding difficulties, all central to bronchiolitis severity [10]. As demonstrated in this study, its ability to predict hospitalization rates underscores its utility in guiding clinical decision-making. For example, children with severe PRESS scores had significantly higher hospitalization rates compared to those with moderate or mild scores: 31 (63.3%) for severe, 18 (36.7%) for moderate, and 0 (0%) for mild. This predictive capacity provides clinicians with a valuable tool for prioritizing care and optimizing resource allocation, especially in settings with limited infrastructure. By addressing the need for a standardized and efficient bedside assessment tool, the PRESS score stands out as a robust alternative to existing scales, capturing the severity of bronchiolitis without compromising on feasibility or accuracy [11].

However, studies comparing hospitalization rates across different severity grades of bronchiolitis based on PRESS scoring are limited. This study addresses this gap by determining the frequency of various bronchiolitis severity levels and comparing hospitalization rates according to PRESS scores, contributing to improved patient management and resource allocation in pediatric care.

## Materials and methods

A prospective descriptive study was performed in the Pediatric Department of a tertiary care hospital in Lahore, Pakistan, for six months, from November 27, 2023, to May 27, 2024, which included the bronchiolitis season in our study setting. The study was approved by the Ethical Review Board of Rawalpindi Medical University (ID-25-49-2023; dated October 17, 2023), and all the parents or guardians of participants gave written informed consent for inclusion in the study, as well as the publication of data. Using the World Health Organization calculator for sample size, a total of 82 samples was calculated by keeping the confidence interval at 95%, the expected proportion of the population as P = 16.3%, and precision as d = 8%. Data were collected using consecutive sampling.

The inclusion criteria were: (1) patients aged one month to two years; (2) all children with bronchiolitis (as diagnosed), defined as the initial acute occurrence of respiratory disorder with tachypnea, wheezing, oxygen saturation below 95%, and feeding difficulty (reported by parents) in children under two years old, with a previous history of low-grade fever (<100°F) and rhinorrhea. The exclusion criteria were: (1) patients with chronic (long-standing) lung and cardiac diseases (congenital heart disease, cystic fibrosis, neuromuscular disease, bronchopulmonary dysplasia); (2) previous episode of respiratory infection requiring intubation; (3) severe feeding difficulties.

Infants under one month were excluded for population uniformity and consistent PRESS score application. This age group is more likely to exhibit atypical symptoms and complications, requiring separate clinical management protocols. Similarly, children with pre-existing conditions, such as congenital heart disease or chronic lung disease, were excluded to avoid confounding the evaluation of the PRESS score, as these comorbidities significantly influence disease severity and outcomes.

Patients who met the selection criteria were enrolled in the study from the emergency unit at our hospital. Demographic details (name, age, sex, and birth weight) were recorded. All baseline respiratory rates were noted; the PRESS score was calculated for each patient as described earlier, and the severity grade was noted. Severity categories based on the PRESS score were operationally defined as follows (Table 1): Mild (0-1): patients typically discharged home with no need for hospitalization. Moderate (2-3): patients admitted to the general pediatric ward for closer observation and supportive care. Severe (4-5): patients requiring admission to the Pediatric Intensive Care Unit (PICU) due to significant respiratory compromise, including oxygen saturation <90%, intercostal recession, and wheezing requiring respiratory support.

**Table 1:** Components and Scoring Criteria of the PRESS System. This table presents the PRESS score components (respiratory rate, wheezing, accessory muscle use, SpO₂, feeding difficulties), with scores assigned between 0 and 1 for each component. The final PRESS score categorization is as follows: Mild (0-1), Moderate (2-3), Severe (4-5). PRESS: Pediatric Respiratory Severity Score

Score Component	Operational Definition		Scoring
Respiratory rate	The respiratory rate is measured when the patient is at rest at room temperature		0 or 1
Wheezing	It is a sharp expiratory sound detected via auscultation		0 or 1
Use of accessory muscle	Observable use of any of the accessory muscles		0 or 1
SpO_2_	When the oxygen saturation is less than 95% on room air		0 or 1
Feeding difficulties	Refusal to feed, prolonged feeding time compared to usual, frequent pauses during feeding, or interruptions requiring caregiver intervention		0 or 1
PRESS score	0-1: categorized as mild, 2-3: categorized as moderate, 4-5: categorized as severe		0-5
Criteria of tachypnea	Month	Respiratory rate	
	<12	60<	1
	12	40<	1
	36	30<	1
	156<	20<	1

Patients with severe respiratory distress requiring intubation were also included in the severe category and managed in the PICU. This stratification was based on the clinical management protocols of the study hospital, which prioritize resource allocation in a resource-limited setting. 

Tachypnea was defined based on age-specific respiratory rate thresholds commonly used in clinical practice and institutional protocols. This approach was chosen for its practical applicability in bedside assessments while ensuring consistency with widely accepted clinical guidelines. Accessory muscle use was defined as any retraction (noticeable) of one or more of the following muscles: suprasternal/sternomastoid, intercostal, and subcostal. Feeding difficulties were defined as refusal to feed, prolonged feeding time compared to usual, frequent pauses during feeding, or interruptions requiring caregiver intervention. Severe feeding difficulties, such as complete refusal to feed or persistent vomiting leading to dehydration or clinical deterioration, were excluded. This definition aligns with established guidelines for assessing feeding difficulties in pediatric respiratory illnesses [11]. Patients were managed per hospital protocol, and data regarding status and hospitalization, etc., were collected after six hours. Considerations for hospitalization included persistent resting oxygen saturation below 90% in room air, dyspnea, wheezing, intercostal recession, and feeding difficulties assessed clinically after six hours.

Descriptive statistics of percentages and frequency were given for qualitative variables, such as gender, hospitalization rate, and PRESS scores. Means with standard deviation were reported for quantitative variables, such as gestational age, respiratory rate, and birth weight. A Pearson Chi-square test was applied to compare the hospitalization rate within the categories of PRESS score, i.e., mild, moderate, and severe. Stratification was used to control confounders, such as gender, weight, and age. The Chi-square test was applied after stratification, and the α-level was set to <0.05. IBM SPSS Statistics for Windows, Version 21 (Released 2012; IBM Corp., Armonk, NY, USA) was used for data analysis.

## Results

The overall characteristics of the sample are summarized in Table 2. The mean age of the children was 13.24 ± 6.65 months, with a range of 1-24 months. Of the total sample, 40 (48.8%) of the children were male.

**Table 2:** Characteristics of the Study Sample. Data is represented as Mean ± SD for continuous variables (age, weight) and N (%) for categorical variables (gender, PRESS score severity, hospitalization status). PRESS: Pediatric Respiratory Severity Score

Characteristic	Mean ± SD/Frequency (N, (%))	Range
Age (months)	13.24 ± 6.65	1 - 24
Gender		
- Male	40 (48.8%)	-
- Female	42 (51.2%)	-
Weight (kg)	7.43 ± 2.61	4 - 15
PRESS Score Severity		
- Mild	22 (26.8%)	-
- Moderate	27 (32.9%)	-
- Severe	33 (40.2%)	-
Hospitalization Status		
- Hospitalized	49 (59.67%)	-
- Not Hospitalized	33 (40.33%)	-

**Figure 1:**
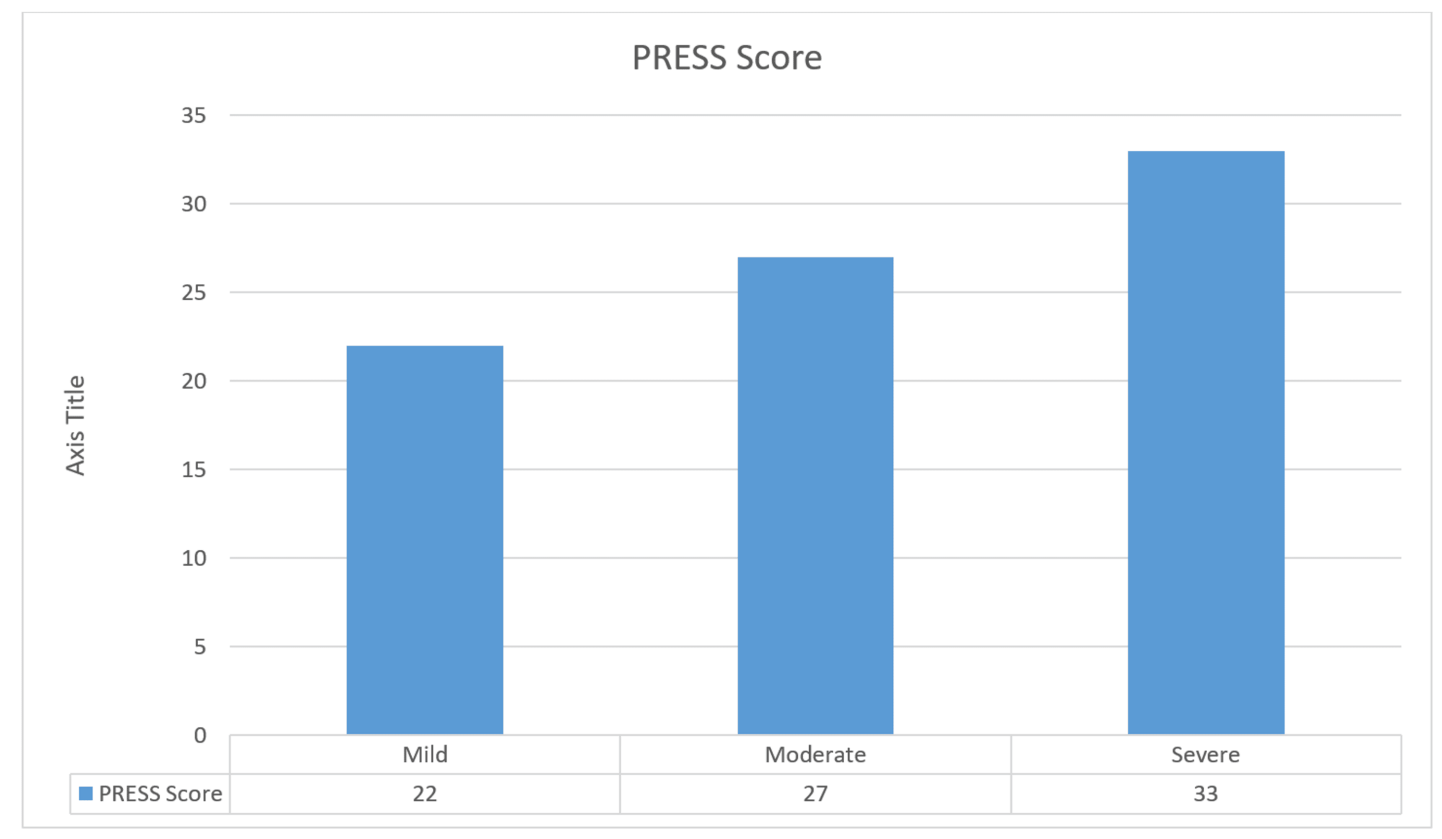
Distribution of PRESS Among Children with Bronchiolitis. This figure presents the distribution of PRESS scores categorized as mild, moderate, and severe, illustrating the frequency of each severity level among the study population. Data is presented as N for each severity category (mild, moderate, severe). PRESS: Pediatric Respiratory Severity Score

The mean weight was 7.43 ± 2.61 kg, with a range of 4 to 15 kg. PRESS scores were mild in 22 (26.8%), moderate in 27 (32.9%), and severe in 33 (40.2%) (Figure 1).

Of the 49 (59.67%) children admitted to the hospital, 18 (36.7%) had moderate PRESS scores and were admitted to the general pediatric ward, while 31 (63.3%) had severe PRESS scores and required intensive care in the PICU. Patients classified as mild were not hospitalized but were discharged home with supportive care instructions. The significant association observed between PRESS scores and hospitalization outcomes (p-value < 0.001) reinforces the utility of this score in predicting the need for escalation of care. Notably, patients requiring intubation were classified as severe, aligning with the study's operational definitions for this category.

PRESS scores strongly correlated with the incidence of hospitalization, with higher scores indicating an increased likelihood of hospitalization (p < 0.001). Of those hospitalized, 31 (63.3%) had severe PRESS scores, and 18 (36.7%) had moderate PRESS scores (Table 3).

**Table 3:** Hospitalization Frequency Across PRESS Score-Based Severity Grades in Pediatric Bronchiolitis. Data is presented as N (%) for each severity category. Statistical analysis was conducted using the Chi-square test (χ²) to compare hospitalization rates across PRESS score groups. A p-value of <0.05 was considered statistically significant. PRESS: Pediatric Respiratory Severity Score

	Hospitalization		Total	χ²	p-value
	Yes	No			
Mild	0 (0%)	22 (66.7%)	22	49.24	<0.001
Moderate	18 (36.7%)	9 (27.3%)	27		
Severe	31 (63.3%)	2 (6.1%)	33		
Total	49	33	82		

The association was significant between hospitalization and PRESS scores in the different age groups (χ² = 52.94, p-value < 0.001) (Table 4).

**Table 4:** Hospitalization Frequency by Age Group and PRESS Score-Based Bronchiolitis Severity. Data is represented as N (%) for each severity category, stratified by age groups. Statistical analysis was performed using the Chi-square test (χ²). A p-value of <0.05 was considered statistically significant. PRESS: Pediatric Respiratory Severity Score

Age Group (Months)	PRESS Score Severity	Hospitalized, N (%)	Not Hospitalized, N (%)	χ²	p-value
2-8	Mild	0 (0%)	5 (38.5%)	52.94	<0.001
	Moderate	8 (50.0%)	2 (15.4%)		
	Severe	8 (50.0%)	0 (0%)		
9-16	Mild	0 (0%)	7 (53.8%)		
	Moderate	4 (23.5%)	5 (38.5%)		
	Severe	13 (76.5%)	0 (0%)		
17-24	Mild	0 (0%)	10 (71.4%)		
	Moderate	6 (37.5%)	2 (14.3%)		
	Severe	10 (62.5%)	2 (14.3%)		

A significant association was found between hospitalization and PRESS scores among male and female patients, i.e., male (p < 0.001) and female (p < 0.001) (Table 5). In all weight categories, hospitalization was significantly associated with the PRESS score (Table 5). Overall, even considering confounders, the PRESS scores are a significant predictor of hospitalization in children.

**Table 5:** Hospitalization Frequency by Sex and Birth Weight Across PRESS Score-Based Bronchiolitis Severity. Data is represented as N (%) for each sex and birth weight category. The Chi-square test (χ²) was used to compare hospitalization rates across different categories. A p-value of <0.05 was considered statistically significant (for the 13-16 kg group, the Chi-square test could not be performed due to insufficient variation in data, mostly zero values). PRESS: Pediatric Respiratory Severity Score

Variable	PRESS Score	Hospitalized (n, %)	Not Hospitalized (n, %)	Total (n)	χ²	p-value
Sex						
Male	Mild	0 (0%)	10 (25.0%)	10	20.8	<0.001
	Moderate	10 (20.4%)	4 (10.0%)	14		
	Severe	14 (28.6%)	2 (5.0%)	16		
Female	Mild	0 (0%)	12 (30.0%)	12	29.23	<0.001
	Moderate	8 (16.3%)	5 (12.5%)	13		
	Severe	17 (34.7%)	0 (0%)	17		
Birth Weight (kg)						
4-8	Mild	0 (0%)	15 (37.5%)	15	44.67	<0.001
	Moderate	14 (28.6%)	3 (7.5%)	17		
	Severe	24 (49.0%)	0 (0%)	24		
9-12	Mild	0 (0%)	7 (17.5%)	7	12.36	0.002
	Moderate	1 (2.0%)	6 (15.0%)	7		
	Severe	7 (14.3%)	2 (5.0%)	9		
13-16	Mild	0 (0%)	0 (0%)	0	N/A	N/A
	Moderate	3 (6.1%)	0 (0%)	3		
	Severe	0 (0%)	0 (0%)	0		

## Discussion

Our study aimed to determine the distribution of bronchiolitis severity using the PRESS and compare hospitalization rates across different severity grades based on PRESS scoring. The primary objective was to evaluate the utility of the PRESS score in guiding admission decisions, distinguishing patients who require hospital care (ward or ICU) from those who can be safely managed at home. While severity stratification traditionally correlates with discharge, ward admission, or PICU admission, the study's operational definitions reflect the clinical context of a resource-limited setting. Severe cases, based on PRESS scores, encompass patients needing intensive monitoring or respiratory support, including intubation, in the PICU. These findings demonstrate that the PRESS score effectively discriminates between the need for ward and ICU admission, even within limited healthcare resources.

The mean age of the children in our cohort was 13.24 ± 6.65 months, with an almost equal gender distribution. While Miyaji et al. reported a higher proportion of boys (60.9%) and an older average age of 27.6 ± 31.8 months, their study included older children and a broader range of respiratory infections beyond bronchiolitis [9]. This difference in study populations limits direct comparisons, but their findings, nonetheless, support the utility of the PRESS score in stratifying respiratory illness severity.

According to recent data from the World Bank, Pakistan has only 0.6 hospital beds per 1,000 people, a figure significantly lower than that of many developed countries, such as the USA, which has approximately 2.8 beds per 1,000 people [12]. This disparity in healthcare resources likely contributes to the stricter hospitalization criteria in Pakistan, particularly for mild and moderate bronchiolitis cases, leading to lower overall hospitalization rates compared to countries with better healthcare infrastructure.

The findings of Miyaji et al. also demonstrated significant differences in respiratory rate, retraction scores, and hospitalization rates across bronchiolitis severity levels [9]. Our study mirrors these results, as we also found significant differences in hospitalization rates across mild, moderate, and severe cases (p-value < 0.001). This highlights the importance of respiratory rate and retractions as reliable indicators of respiratory distress, which are integral to assessing the severity of bronchiolitis and guiding clinical decisions regarding hospitalization and management. These symptoms are critical components of the PRESS score, highlighting their utility in predicting clinical outcomes. Our study suggests that children with severe bronchiolitis, as classified by the PRESS score, were more frequently hospitalized. This supports its utility as a tool for identifying cases requiring more intensive management and extended hospital stays. Using severity scales like PRESS may also assist clinicians in predicting the length of hospital stay and guiding the treatment process.

The study by Rodriguez et al. highlighted the clinical relevance of the PRESS score, confirming its utility not only for clinical decision-making but also for assessing treatment effectiveness in routine practice and clinical trials [11]. This aligns with the guidelines provided by the American Academy of Pediatrics (AAP) on diagnosing and managing bronchiolitis. The AAP guidelines advise against the routine use of bronchodilators but suggest that, in select cases, nebulized bronchodilators may be administered, with objective pre- and post-therapy assessments, such as those provided by the PRESS score, used to evaluate treatment responses [1].

It is important to note that bronchiolitis severity scores differ significantly in the criteria they assess. Some focus solely on clinical signs of respiratory distress, such as retractions and wheezing, while others incorporate additional measures, such as general appearance, cyanosis, and oxygen saturation [1]. Our study demonstrated that children classified as having severe bronchiolitis, according to the PRESS score, were more frequently hospitalized, reinforcing the score's value in predicting severe outcomes. This value is particularly relevant in resource-limited settings like Pakistan, where identifying high-risk cases can improve resource allocation and ensure appropriate care. Thus, the PRESS score enhances patient care in real time and supports systematic improvements in resource-limited healthcare systems by providing a reliable method for stratifying bronchiolitis severity.

The Modified Tal Score (MTS) represents a highly accurate test, even if it is not user-friendly and needs more time [13]. The PRESS scale is easy to use and provides results very quickly. This makes it a perfect fit for emergency rooms. The BROSJOD assesses the severity according to wheezing, indrawing, airway entry, respiratory rate, heart rate, and oxygen saturation [8]. While BROSJOD offers useful data for refractory cases because it can cover the respiratory aspects, the PRESS score notes dysphagia, which is important and can be applied faster, thus increasing clinicians' efficiency [14]. The RDAI estimates airway obstruction with wheezing, retractions, and respiratory rate [15]. Although RDAI is consistent with our observation of both the respiratory rate and retractions as indicators of hospitalization, it does not incorporate oxygen saturation, a relevant parameter to evaluate bronchiolitis severity. Severity scoring tools, such as PRESS, MTS, BROSJOD, and RDAI, facilitate quick severity scoring and inform management decisions. PRESS is noteworthy due to its simplicity and applicability in resource-limited settings. It is a critical, cost-effective triage and resource allocation tool for high-volume or limited healthcare scenarios.

This study has several limitations that should be considered when interpreting the results. First, the small sample size limits the statistical power of the findings and may affect the ability to detect subtle differences between groups. Second, the study was conducted at a single center, which, combined with the limited sample size, restricts the generalizability of the results to other populations or healthcare settings. Third, the data collection period (November to May) was relatively short and did not cover the peak bronchiolitis season in Pakistan (July to September), which may impact the applicability of the findings to the period of highest disease incidence. Fourth, the study was conducted in a resource-limited country, where healthcare infrastructure and hospitalization criteria may differ significantly from those in high-income countries. These contextual factors further limit the generalizability of the findings to other regions.

Additionally, the use of non-probability consecutive sampling may have introduced selection bias, potentially limiting the representativeness of the study population. A randomized sampling approach would have minimized this bias and provided a more representative understanding of bronchiolitis severity across different demographics. Furthermore, while the PRESS is useful for assessing respiratory illness severity, it was not initially developed for bronchiolitis. This may influence the accuracy of severity categorization, as certain clinical nuances specific to bronchiolitis, such as viral etiologies and seasonality, may not be fully captured. The exclusion of patients younger than one month and those with serious comorbidities also represents a limitation, as these groups are at higher risk for severe bronchiolitis. Their inclusion, however, may have introduced confounding variables affecting the PRESS score’s utility. Finally, the PRESS score relies on clinical features such as respiratory rate, wheezing, and accessory muscle use, which, while indicative, may vary in their sensitivity to subtle differences in bronchiolitis severity. Future studies should validate the PRESS score in high-risk populations and across multiple bronchiolitis seasons to assess its broader applicability and improve predictive accuracy for hospitalization needs.

## Conclusions

In conclusion, our study suggests that PRESS is a useful tool for classifying the severity of bronchiolitis and identifying patients at increased risk of hospitalization. The study found that children in the severe category, as determined by the PRESS score, had the highest frequency of bronchiolitis severity and correspondingly higher hospitalization rates. These findings highlight the potential of the PRESS score to guide clinical decision-making and optimize resource allocation, particularly in healthcare systems with limited infrastructure. However, given the limitations of this study, the results should be interpreted with caution. To establish its broader applicability, further research is needed to validate the PRESS score in diverse populations and healthcare settings.

## References

[1] Ralston SL, Lieberthal AS, Meissner HC (2014). Clinical practice guideline: the diagnosis, management, and prevention of bronchiolitis. Pediatrics.

[2] Sharif H, Jan SS, Sharif S, Seemi T, Naeem H, Rehman J (2022). Respiratory diseases' burden in children and adolescents of marginalized population: a retrospective study in slum area of Karachi, Pakistan. Front Epidemiol.

[3] Zorc JJ, Hall CB (2010). Bronchiolitis: recent evidence on diagnosis and management. Pediatrics.

[4] Nagakumar P, Doull I (2012). Current therapy for bronchiolitis. Arch Dis Child.

[5] Dayan PS, Roskind CG, Levine DA, Kuppermann N (2004). Controversies in the management of children with bronchiolitis. Clin Pediatr Emerg Med.

[6] McCallum GB, Morris PS, Wilson CC, Versteegh LA, Ward LM, Chatfield MD, Chang AB (2013). Severity scoring systems: are they internally valid, reliable and predictive of oxygen use in children with acute bronchiolitis?. Pediatr Pulmonol.

[7] Gonzales R, Bartlett JG, Besser RE, Cooper RJ, Hickner JM, Hoffman JR, Sande MA (2001). Principles of appropriate antibiotic use for treatment of uncomplicated acute bronchitis: background. Ann Intern Med.

[8] Balaguer M, Alejandre C, Vila D, Esteban E, Carrasco JL, Cambra FJ, Jordan I (2017). Bronchiolitis Score of Sant Joan de Déu: BROSJOD score, validation and usefulness. Pediatr Pulmonol.

[9] Miyaji Y, Sugai K, Nozawa A (2015). Pediatric respiratory severity score (PRESS) for respiratory tract infections in children. Austin Virol Retrovirol.

[10] Bressan S, Balzani M, Krauss B, Pettenazzo A, Zanconato S, Baraldi E (2013). High-flow nasal cannula oxygen for bronchiolitis in a pediatric ward: a pilot study. Eur J Pediatr.

[11] Rodriguez H, Hartert TV, Gebretsadik T, Carroll KN, Larkin EK (2016). A simple respiratory severity score that may be used in evaluation of acute respiratory infection. BMC Res Notes.

[12] (2025). Hospital beds (per 1,000 people). https://data.worldbank.org/indicator/SH.MED.BEDS.ZS.

[13] Golan-Tripto I, Goldbart A, Akel K, Dizitzer Y, Novack V, Tal A (2018). Modified Tal Score: validated score for prediction of bronchiolitis severity. Pediatr Pulmonol.

[14] Tutor JD (2020). Dysphagia and chronic pulmonary aspiration in children. Pediatr Rev.

[15] Destino L, Weisgerber MC, Soung P (2012). Validity of respiratory scores in bronchiolitis. Hosp Pediatr.

